# New High-Quality Draft Genome of the Brown Rot Fungal Pathogen *Monilinia fructicola*

**DOI:** 10.1093/gbe/evz207

**Published:** 2019-09-27

**Authors:** Rita Milvia De Miccolis Angelini, Gianfranco Romanazzi, Stefania Pollastro, Caterina Rotolo, Francesco Faretra, Lucia Landi

**Affiliations:** 1 Department of Soil, Plant and Food Sciences, University of Bari ‘Aldo Moro’, Italy; 2 Department of Agricultural, Food and Environmental Sciences, Marche Polytechnic University, Ancona, Italy

**Keywords:** brown rot, de novo assembly, genome annotation, next-generation sequencing, third-generation sequencing, stone fruit

## Abstract

Brown rot is a worldwide fungal disease of stone and pome fruit that is caused by several *Monilinia* species. Among these, *Monilinia fructicola* can cause severe preharvest and postharvest losses, especially for stone fruit. Here, we present a high-quality draft genome assembly of *M. fructicola* Mfrc123 strain obtained using both Illumina and PacBio sequencing technologies. The genome assembly comprised 20 scaffolds, including 29 telomere sequences at both ends of 10 scaffolds, and at a single end of 9 scaffolds. The total length was 44.05 Mb, with a scaffold N50 of 2,592 kb. Annotation of the *M. fructicola* assembly identified a total of 12,118 genes and 13,749 proteins that were functionally annotated. This newly generated reference genome is expected to significantly contribute to comparative analysis of genome biology and evolution within *Monilinia* species.

## Introduction

*Monilinia fructicola* (phylum *Ascomycota*, family *Sclerotiniaceae*) is one of most important causal agents of brown rot, which is one of the main diseases of pome and stone fruit. Brown rot can cause severe yield losses during both field production and postharvest processing (Mari et al. 2012; Karaca et al. 2014; Oliveira Lino et al. 2016; Abate, Pastore, et al. 2018). Several species of the *Monilinia* genus are responsible for this disease, including *Monilinia laxa* and *Monilinia fructigena* (Villarino et al. 2013; Rungjindamai et al. 2014), although *M. fructicola* is considered the most destructive of these pathogens on stone fruit. Indeed, *M. fructicola* is a quarantine pathogen in the European Union and is included in List A2 of the European and Mediterranean Plant Protection Organisation (https://www.eppo.int/ACTIVITIES/plant_quarantine/A2_list; last accessed October 1, 2019). *Moniliniafructicola* was introduced into Europe in 2001 (Lichou et al. 2002), and then rapidly spread and became prevalent over the former indigenous species *M. laxa* and *M. fructigena* (CABI 2018; Abate, Pastore, et al. 2018). This fungus overwinters on mummified fruit or in infected plant tissues. In spring, conidia are produced under moist conditions, with wind dispersal, and infection of blossoms, causing blossom blight. However, the most severe yield losses occur in the postharvest phase, during fruit storage and transport (Feliziani et al. 2013; Martini and Mari 2014).

The development of high-throughput sequencing technologies is quickly improving biological studies on fungal pathogens (Shendure et al. 2017). Whole genome knowledge provides a complete overview in terms of their potential virulence mechanisms, with the aim being to design improved disease-control approaches (Möller and Stukenbrock 2017). The availability of complete genomic resources can be very useful in the study of evolutionary dynamics and genetic adaptions to highly diverse environments (Mitchell-Olds et al. 2007). The availability of complete genomic data of *M. fructicola* gives the opportunity to the scientific community to perform studies aimed at exploring the population biology of the pathogen in more detail and its interactions with host plants (Dowling et al. 2019). This should be of help for an understanding of the reasons underlying the fitness of the pathogen and its rapid spread to new geographical areas. For these reasons, we have generated a high-quality draft genome and annotated protein-coding genes of *M. fructicola* that represents a great improvement over the draft genomes already available in public repositories, which are highly fragmented with no gene annotation (Rivera et al. 2018). A hybrid and hierarchical de novo assembly strategy was used to obtain the genome of the *M. fructicola* Mfrc123 strain through a combination of Illumina short reads next-generation sequencing, and Pacific Biosciences (PacBio) long reads third-generation sequencing, as previously reported for de novo assembly of the *M. fructigena* genome (Landi et al. 2018).

## Materials and Methods

### Sample Collection, Library Construction, and Sequencing

The monoconidial Mfrc123 strain (CBS 144850) of *M. fructicola* (NCBI:txid38448; supplementary fig. S1, Supplementary Material online) was isolated from cherry (*Prunus avium*) in 2014 during monitoring of *Monilinia* populations in southern Italy (Abate, Pastore, et al. 2018). The strain was grown in potato dextrose broth (infusion from 200 g peeled and sliced potatoes kept for 1 h at 60 °C, with 20 g dextrose, adjusted to pH 6.5, per liter) for 48 h at 25 ± 1 °C, in darkness and under shaking (150 rpm). The strain was characterized at both the phenotypic and molecular levels (Abate, De Miccolis Angelini, et al. 2018; Abate, Pastore, et al. 2018; De Miccolis Angelini et al. 2018). Genomic DNA was extracted using Gentra Puregene tissue kits (Qiagen, Milan, Italy), according to the manufacturer instructions. The genomic DNA quality and quantity were determined using a Nanodrop 2000 spectrophotometer (Thermo Fisher Scientific Inc., Wilmington, DE) and a Qubit 2.0 fluorimeter (Life Technologies Ltd., Paisley, UK). DNA integrity was analyzed (Bioanalyser 2100; Agilent Technologies, Santa Clara, CA). Genome sequencing for short 2× 100-bp paired-end reads (HiSeq 2500 platform; Illumina Sequencing Technology) and long 20-kb reads (RSII platform; PacBio Sequencing Technology) were both performed by an external service (Macrogen Inc., Next-Generation Sequencing Service, Geumcheon-gu, Seoul, South Korea). The Illumina short reads were analyzed for quality using FastX-tools and trimmed with the Trimmomatic (version 0.36) software (Bolger et al. 2014) with the following parameters: 1) LEADING and TRAILING = 3, which removes bases from the two ends of the reads if they are below a threshold quality of 3, 2) SLIDING WINDOW = 4:2, which cuts the reads when the average quality within the window composed of four bases falls below a threshold of 2, and 3) MINLEN = 50, which removes the reads shorter than 50 bp. Adaptor-free PacBio subreads were used for the genome assembly.

### De Novo Assembly of the Genome

The genome of *M. fructicola* Mfrc123 strain was assembled by a hybrid and hierarchical assembly strategy, using all the reads from both the Illumina and PacBio sequencing to produce scaffolds through the DBG2OLC pipeline available from https://github.com/yechengxi/DBG2OLC; last accessed October 1, 2019 (Ye et al. 2016). SparseAssembler (Ye et al. 2012; https://github.com/yechengxi/SparseAssembler; last accessed October 1, 2019) was used to preassemble the cleaned Illumina short reads into contigs, using the following parameters: LD, 0; NodeCovTh, 1; EdgeCovTh, 0; k, 61; g, 15; and PathCovTh, 100, GS 50000000. The overlap and layout were performed with the DBG2OLC module (Ye et al. 2016; https://github.com/yechengxi/DBG2OLC; last accessed October 1, 2019) on the output contigs from SparseAssembler and the PacBio long subreads, to construct the assembly backbone from the best overlaps of reads using the following parameters: AdaptiveTh, 0.001; KmerCovTh, 2; and MiniOverlap, 20. In the final step, all of the related reads were aligned to each backbone using BLASR (Chaisson and Tesler 2012; https://github.com/PacificBiosciences/blasr; last accessed October 1, 2019), and the most likely sequence was calculated as the polished assembly using the consensus module Sparc (Ye and Ma 2016; https://github.com/yechengxi/Sparc; last accessed October 1, 2019), both with the default settings.

The quality assessment of the draft assembly was performed by mapping RNA-Seq reads from the same *M. fructicola* Mfrc123 strain (De Miccolis Angelini et al. 2018) using the CLC Genomics Workbench v. 7.0.3 software (CLC Bio, Aarhus, Denmark), with the default parameters. Unmapped reads were then assembled using the CLC de novo assembler, and the contigs obtained were submitted to BlastN searches against the GenBank nucleotide collection (downloaded, January 10, 2018) with local BLAST+ v. 2.3.0 (Camacho et al. 2009), setting the *E*-value cutoff at 10^−3^. Ribosomal DNA sequences were identified and BlastN aligned to the PacBio subreads. The subread -s1p0/78570/021991RQ = 0.891 was selected for the best homology and used as reference for the Illumina reads alignment using the SeqMan NGen software (Lasergene v. 15.0.1; DNASTAR Inc., Madison, WI) to improve the sequence accuracy. Here, we obtained a 28,000-bp scaffold made up of three rDNA repeat unit sequences. Benchmarking Universal Single-Copy Orthologs (BUSCO v. 3.0.2) (Simão et al. 2015; http://gitlab.com/ezlab/busco; last accessed July 30, 2019) was used with the default parameters to evaluate the completeness of the de novo genome assembly based on the 290 BUSCO groups in the fungi_odb9 lineage data set (http://busco.ezlab.org/; last accessed July 30, 2019) and *Botrytiscinerea* as the reference species for gene finding with Augustus v. 3.3.2 (http://bioinf.uni-greifswald.de/augustus/; last accessed July 30, 2019).

### Repeats Annotation

Simple repeats and low complexity elements were searched using Repeat Masking (http://www.repeatmasker.org/cgi-bin/WEBRepeatMasker; last accessed July 30, 2019) against the Repbase-derived RepeatMasker library of repetitive elements, with the default parameters. Telomere sequences were identified by searching both ends of the scaffolds for high copy number repeats using BlastN searches and manual inspection.

### Protein-Coding Gene Prediction and Functional Annotation

Gene prediction was performed with Augustus implemented in the Blast2GO PRO package (v.5.2.5) using *B. cinerea* as the model species and the RNA-Seq reads as a guide. Blast2GO PRO was used to obtain gene functional classification based on Gene Ontology (GO) terms in the “biological process,” “molecular function,” and “cellular component” categories.

## Results and Discussion

### Library Construction and Sequencing

A total of 11,014 Mb of Illumina reads (109,054,254 total reads with 90.16% of bases with quality score >30) were generated with an estimated 250× average depth of sequencing coverage (http://identifiers.org/ncbi/insdc.sra:SRR8599895). A total of 1,057-Mb PacBio long reads (polymerase read N50 of 19,107, quality of 0.858; average subread length of 9.8 kb and subread N50 length of 14.34 kb) were generated with an estimated 24× average depth of sequencing coverage (http://identifiers.org/ncbi/insdc.sra:SRR8608008).

### De Novo Assembly of the Genome

A complete draft genome without gaps of 44.05 Mb was obtained with a scaffold N50 of 2,592.8 kb (table 1). It was assembled in 16 large scaffolds (from 4.24 to 1.89 Mb; table 2), three very small scaffolds (from 0.36 to 0.26 Mb), and one scaffold made up of three repeat unit sequences of ribosomal DNA. Both telomeric sequences were detected in half of the large scaffolds, with only one telomere in the remaining half (table 2). The number of large scaffolds reconstructed in the *M. fructicola* Mfrc123 strain corresponded to the 16 large chromosomes of the closely related species *B.cinerea* and *Sclerotinia sclerotiorum* (Amselem et al. 2011; Derbyshire et al. 2017). This is in accordance with the short phylogenetic distances existing among these species within the *Sclerotiniaceae* family (Amselem et al. 2011).

**Table 1:** Comparison among the De Novo Genome Assembly Results of *Monilinia fructicola* Strains Mfrc123 (This Study), BR-32 (GCA_002909715.1), and LMK125 (GCA_002162545.1)

Feature	**Assembly** ^a^		
	Mfrc123	BR-32	LMK125
Genome size in scaffolds (bp)	44,047,900	42,820,478	44,684,193
Total ungapped length (bp)	44,047,900	42,800,193	43,575,363
GC content (%)	40.79	41.72	40.12
Number of scaffolds	20	2,349	846
Maximum scaffold length (bp)	4,238,823	414,517	3,180,511
Minimum scaffold length (bp)	28,364	426	200
Mean scaffold size (bp)	2,202,395	18,229	52,818
Scaffold N50	2,592,823	66,420	992,265
Scaffold N90	1,968,927	10,383	304,023
Scaffold L50	7	188	15
Number of genes	12,118	NA	NA
Number of CDS	13,749	NA	NA
Proteins with no BLAST hits	1,679	NA	NA
Proteins with BLAST hits	2,078	NA	NA
Proteins with mapping	655	NA	NA
Proteins with GO annotation	8,456	NA	NA
Total proteins with significant hits (%)	11,397 (82.9)	NA	NA

a NA, not available.

**Table 2:** Statistics for Scaffolds Obtained from the *Monilinia fructicola* Mfrc123 Strain Genome Assembly

Name	Length (bp)	GC (%)	Number of Telomeric Repeats		Number of Repeated Elements (%)		Number of CDS	Number of Genes
			Start (3′CCCTAA5′)	End (5′TTAGGG3′)	Simple Repeats	Low Complexity Regions		
Scaffold_001	4,238,823	42.36	NA^a^	15	3,213 (4.10)	285 (0.42)	1,467	1,259
Scaffold_002	3,728,272	42.06	23	14	2,773 (4.02)	249 (0.42)	1,275	1,109
Scaffold_003	3,609,855	41.58	19	23	2,836 (4.06)	260 (0.40)	1,225	1,077
Scaffold_004	3,332,839	41.50	18	19	2,461 (3.79)	210 (0.35)	1,151	999
Scaffold_005	2,656,653	42.11	NA	17	2,063 (4.25)	197 (0.41)	866	765
Scaffold_006	2,602,481	40.60	NA	20	1,823 (3.90)	163 (0.35)	765	682
Scaffold_007	2,592,823	41.30	17	18	1,742 (3.68)	150 (0.32)	809	723
Scaffold_008	2,576,762	40.99	NA	24	2,047 (4.46)	185 (0.38)	782	686
Scaffold_009	2,530,796	41.34	18	NA	2,160 (4.48)	224 (0.49)	734	666
Scaffold_010	2,442,597	40.83	19	24	2,192 (4.73)	193 (0.45)	774	675
Scaffold_011	2,335,009	41.74	NA	18	1,754 (3.94)	162 (0.41)	753	650
Scaffold_012	2,285,153	40.66	21	9	1,783 (4.30)	174 (0.45)	699	623
Scaffold_013	2,201,684	41.88	NA	13	1,651 (3.87)	0.38 (156)	631	566
Scaffold_014	2,106,460	40.87	15	NA	1,794 (4.50)	167 (0.44)	662	591
Scaffold_015	1,968,927	39.71	18	18	1,560 (4.43)	141 (0.45)	583	522
Scaffold_016	1,888,073	39.21	17	14	1,461 (4.29)	136 (0.45)	553	506
Scaffold_017	355,621	15.89	16	15	167 (3.45)	34 (0.54)	3	2
Scaffold_018	311,593	15.56	NA	19	133 (3.18)	19 (0.33)	6	6
Scaffold_019	255,115	19.02	NA	19	135 (4.21)	23 (0.46)	11	11
Scaffold_020 (rDNA)	28,364	42.77	NA	NA	3 (0.45)	3 (0.66)	NA	NA
Total	44,047,900	40.79	—	—	3,131 (0.41)	33,751 (4.13)	13,749	12,118

a NA, not available.

Data obtained from genome completeness analysis by BUSCO showed that 88% of the predicted genes on the assembled genomic sequences were complete and single copy, and only a few were fragmented (10%) or missing (2%) BUSCO orthologs. RNA-Seq reads from the same *M. fructicola* Mfrc123 strain (De Miccolis Angelini et al. 2018) were mapped on the draft assembled genome, and about 82% of them mapped in pairs, whereas 10% of them mapped in broken pairs on the final genome draft version.

### Repeats Annotation

The genome showed 33,751 elements (4.13% of the genome size) that were identified as single repeats, whereas 3,131 elements (0.41% of the genome size) were recognized as low complexity regions (table 2). Our assembly captured a total of 29 stretches of telomeric sequences (5′-TTAGGG-3′) at both ends of 10 scaffolds and at a single end of 9 scaffolds, with repeat numbers ranging from 9 to 23 (table 2), suggesting a good integrity of the assembled genome.

### Protein-Coding Gene Prediction and Functional Annotation

A total of 12,118 genes coding for 13,749 predicted proteins were identified and functionally annotated (tables 1 and 2). In detail, 11,397 proteins (82.9%) had significant BlastP hits, and 8,456 proteins (61.5%) had GO annotation (table 1). According to generic terms distribution at level 3 in Blast2GO, 14 biological processes were identified. The most represented terms were “organic substance metabolic process” (16%), “cellular metabolic process” (15%), “primary metabolic process” (15%), and “nitrogen compound metabolic process” (13%). Among the 11 GO terms identified for molecular functions, the majority were constituted by the following GO terms: “organic cyclic compound binding” (15%), “heterocyclic compound binding” (15%), “ion binding” (14%), “hydrolase activity” (11%), and “transferase activity” (9%). For “cellular component,” a total of eight GO terms were identified, and among these the most represented were “intracellular” (20%), “intracellular part” (20%), “intracellular organelle” (16%), “membrane-bound organelle” (14%), and “intrinsic component of membrane” (12%) (supplementary fig. S2, Supplementary Material online).

## Conclusion

The hybrid and hierarchical assembly strategy, using both Illumina and PacBio sequencing technologies, was successful to produce a new genomic draft of *M. fructicola* Mfrc123 strain that greatly improved those of other strains of the same fungus available in GenBank (BR-32 strain, GCA_002909715.1; and LMK125 strain, GCA_002162545.1) in terms of contiguity (scaffold N50 values), gap-free sequences, and read mappability, also providing a structural and functional annotation of the genome.

The genome assembly has size and number of genes comparable to other annotated genomes of fungi belonging to the *Sclerotiniaceae* family available in public databases, such as *B.cinerea* strains B05.10 (GCA_000143535.4; van Kan et al. 2017), T4 (GCA_000292645.1; Staats and van Kan 2012) and BcDW1 (GCA_000349525.1; Blanco-Ulate et al. 2013), *M. fructigena* strains Mfrg269 (GCA_003260565.1; Landi et al. 2018), *Sclerotinia borealis* strain F-4128 (GCA_000503235.1; Mardanov et al. 2014), and *S.* *sclerotiorum* strain 1980 UF-70 (GCA_001857865.1; Derbyshire et al. 2017). Moreover, the number of large scaffolds reconstructed in the *M. fructicola* Mfrc123 strain corresponds to the 16 large chromosomes of the closely related species *B. cinerea* and *S. sclerotiorum* (Amselem et al. 2011; Derbyshire et al. 2017).

The new genomic resource will allow to gain deeper knowledge on the genome biology of *M. fructicola* and provides valuable information for further evolutionary studies within the *Monilinia* genus and the *Sclerotiniaceae* family, which contain phytopathogenic fungi of great economical relevance worldwide.

## Data Availability

The final genome assembly (GBK) including the annotated genes, mRNAs, and CDSs, RepeatMasker output (GFF) with the repetitive elements coordinates and masked genomic sequences (FASTA), as well as protein sequences (FASTA) and their functional annotations (XLSX) are uploaded into Figshare (https://doi.org/10.6084/m9.figshare.9423230).

## Supplementary Material

Supplementary data are available at *Genome Biology and Evolution* online.

## Supplementary Material

evz207_Supplementary_Data

## Notes

**Data deposition:** The data generated in this study are publicly available from the NCBI/GenBank database at Bioproject ID PRJNA523200. Raw reads from both the Illumina and PacBio sequencing have been deposited in the Sequence Read Archive under accession numbers SRR8599895 and SRR8608008, respectively. This project has been deposited at DDBJ/EMBL/GenBank under the accession VICG00000000. The version described in this article is VICG01000000.

## References

[evz207-B1] Abate D, De Miccolis Angelini RM, Rotolo C, Pollastro S, Faretra F. 2018 Mating system in the brown rot pathogens *Monilinia fructicola*, *Monilinia laxa* and *Monilinia fructigena*. Phytopathology 108(11):1315–1325. 29767553 10.1094/PHYTO-03-18-0074-R

[evz207-B2] Abate D, Pastore C, et al 2018 Characterization of *Monilinia* spp. populations on stone fruits in south Italy. Plant Dis. 102(9):1708–1717. 30125154 10.1094/PDIS-08-17-1314-RE

[evz207-B3] Amselem J, et al 2011 Genomic analysis of the necrotrophic fungal pathogens *Sclerotinia sclerotiorum* and *Botrytis cinerea*. PLoS Genet. 7(8):e1002230. 21876677 10.1371/journal.pgen.1002230 PMC3158057

[evz207-B4] Blanco-Ulate B, Allen G, Powell AL, Cantu D. 2013 Draft genome sequence of *Botrytis cinerea* BcDW1, inoculum for noble rot of grape berries. Genome Announc. 1:e00252-13. 10.1128/genomeA.00252-13 PMC3662820 23704180

[evz207-B5] Bolger AM, Lohse M, Usadel B. 2014 Trimmomatic: a flexible trimmer for Illumina sequence data. Bioinformatics 30(15):2114–2120. 24695404 10.1093/bioinformatics/btu170 PMC4103590

[evz207-B6] Camacho C, et al 2009 BLAST+: architecture and applications. BMC Bioinformatics 10(1):421. 20003500 10.1186/1471-2105-10-421 PMC2803857

[evz207-B7] Chaisson MJ, Tesler G. 2012 Mapping single molecule sequencing reads using basic local alignment with successive refinement (BLASR): application and theory. BMC Bioinformatics 13(1):238. 22988817 10.1186/1471-2105-13-238 PMC3572422

[evz207-B8] CABI, 2018. *Monilinia fructicola* (brown rot). [Datasheet: Pest]. Invasive Species Compendium. Wallingford (United Kingdom): CABI. Available from: https://www.cabi.org/isc/datasheet/34746; last accessed October 1, 2019.

[evz207-B9] De Miccolis Angelini RM, et al 2018 *De novo* assembly and comparative transcriptome analysis of *Monilinia fructicola, Monilinia laxa* and *Monilinia fructigena*, the causal agents of brown rot on stone fruits. BMC Genomics. 19(1):436. 29866047 10.1186/s12864-018-4817-4 PMC5987419

[evz207-B11] Derbyshire M, et al 2017 The complete genome sequence of the phytopathogenic fungus *Sclerotinia sclerotiorum* reveals insights into the genome architecture of broad host range pathogens. Genome Biol Evol. 9(3):593–618. 10.1093/gbe/evx030 PMC5381539 28204478

[evz207-B12] Dowling ME, et al 2019 Preservation of *Monilinia fructicola* genotype diversity within fungal cankers. Plant Dis. 103(3):526–530. 30657426 10.1094/PDIS-05-18-0800-RE

[evz207-B13] Feliziani E, Santini M, Landi L, Romanazzi G. 2013 Pre and postharvest treatment with alternatives to synthetic fungicides to control postharvest decay of sweet cherry. Postharvest Biol Technol. 78:133–138.

[evz207-B14] Karaca H, Pérez-Gago MB, Taberner V, Palou L. 2014 Evaluating food additives as antifungal agents against *Monilinia fructicola in vitro* and in hydroxypropyl methylcellulose–lipid composite edible coatings for plums. Int J Food Microbiol. 179:72–79. 24742996 10.1016/j.ijfoodmicro.2014.03.027

[evz207-B15] Landi L, et al 2018 Genome sequence of the brown rot fungal pathogen *Monilinia fructigena*. BMC Res Notes 11(1):758. 30352625 10.1186/s13104-018-3854-z PMC6199720

[evz207-B16] Lichou J, et al 2002 Une nouvelle moniliose. Phytoma 547:22–25.

[evz207-B17] Mardanov AV, Beletsky AV, Kadnikov VV, Ignatov AN, Ravin NV. 2014 Draft genome sequence of *Sclerotinia borealis*, a psychrophilic plant pathogenic fungus. Genome Announc. 2:e01175-13. 10.1128/genomeA.01175-13 PMC3900894 24459262

[evz207-B18] Mari M, Martini C, Guidarelli M, Neri F. 2012 Postharvest biocontrol of *Monilinia laxa*, *Monilinia fructicola* and *Monilinia fructigena* on stone fruit by two *Aureobasidium pullulans* strains. Biol Control. 60(2):132–140.

[evz207-B19] Martini C, Mari M. 2014 *Monilinia fructicola*, *Monilinia laxa* (Monilinia rot, brown rot) In: Bautista-Baños S, editor. Postharvest decay. Control strategies. Eastbourne (United Kingdom): Academic Press/Elsevier p. 233–265.

[evz207-B20] Mitchell-Olds T, Willis JH, Goldstein DB. 2007 Which evolutionary processes influence natural genetic variation for phenotypic traits? Nat Rev Genet. 8(11):845–856. 17943192 10.1038/nrg2207

[evz207-B21] Möller M, Stukenbrock EH. 2017 Evolution and genome architecture in fungal plant pathogens. Nat Rev Microbiol. 15(12):756–771. 29123226 10.1038/nrmicro.2017.143

[evz207-B22] Oliveira Lino L, et al 2016 Brown rot strikes *Prunus* fruit: an ancient fight almost always lost. J Agric Food Chem. 64(20):4029–4047. 27133976 10.1021/acs.jafc.6b00104

[evz207-B23] Rivera Y, et al 2018 Draft genome resources for the phytopathogenic fungi *Monilinia fructicola*, *M. fructigena*, *M. polystroma*, and *M. laxa*, the causal agents of brown rot. Phytopathology 108(10):1141–1142. 29723113 10.1094/PHYTO-12-17-0418-A

[evz207-B24] Rungjindamai N, Jeffries P, Xu XM. 2014 Epidemiology and management of brown rot on stone fruit caused by *Monilinia laxa*. Eur J Plant Pathol. 140(1):1–17.

[evz207-B25] Shendure J, et al 2017 DNA sequencing at 40: past, present and future. Nature 550(7676):345–353. 29019985 10.1038/nature24286

[evz207-B26] Simão FA, Waterhouse RM, Ioannidis P, Kriventseva EV, Zdobnov EM. 2015 BUSCO: assessing genome assembly and annotation completeness with single-copy orthologs. Bioinformatics 31(19):3210–3212. 26059717 10.1093/bioinformatics/btv351

[evz207-B27] Staats M, van Kan JA. 2012 Genome update of *Botrytis cinerea* strains B05.10 and T4. Eukaryot Cell 11(11):1413–1414. 23104368 10.1128/EC.00164-12 PMC3486023

[evz207-B28] Van Kan JA, et al 2017 A gapless genome sequence of the fungus *Botrytis cinerea*. Mol Plant Pathol. 18(1):75–89. 26913498 10.1111/mpp.12384 PMC6638203

[evz207-B29] Villarino M, et al 2013 Occurrence of *Monilinia laxa* and *M. fructigena* after introduction of *M. fructicola* in peach orchards in Spain. Eur J Plant Pathol. 137(4):835–845.

[evz207-B30] Ye C, Hill CM, Wu S, Ruan J, Ma Z. 2016 DBG2OLC: efficient assembly of large genomes using long erroneous reads of the third-generation sequencing technologies. Sci Rep. 6:1–9. 27573208 10.1038/srep31900 PMC5004134

[evz207-B31] Ye C, Ma ZS. 2016 Sparc: a sparsity-based consensus algorithm for long erroneous sequencing reads. PeerJ 4:e2016. 27330851 10.7717/peerj.2016 PMC4906657

[evz207-B32] Ye C, Ma ZS, Cannon CH, Pop M, Yu DW. 2012 Exploiting sparseness in *de-novo* genome assembly. BMC Bioinformatics 13(S6):S1. 10.1186/1471-2105-13-S6-S1 PMC3369186 22537038

