# Supplementary figures and images for: New High-Quality Draft Genome of the Brown Rot Fungal Pathogen Monilinia fructicola
Source: Genome Biol Evol. 2019 Sep 27;11(10):2850–5. doi: 10.1093/gbe/evz207 (PMC6795239; doi:10.1093/gbe/evz207)

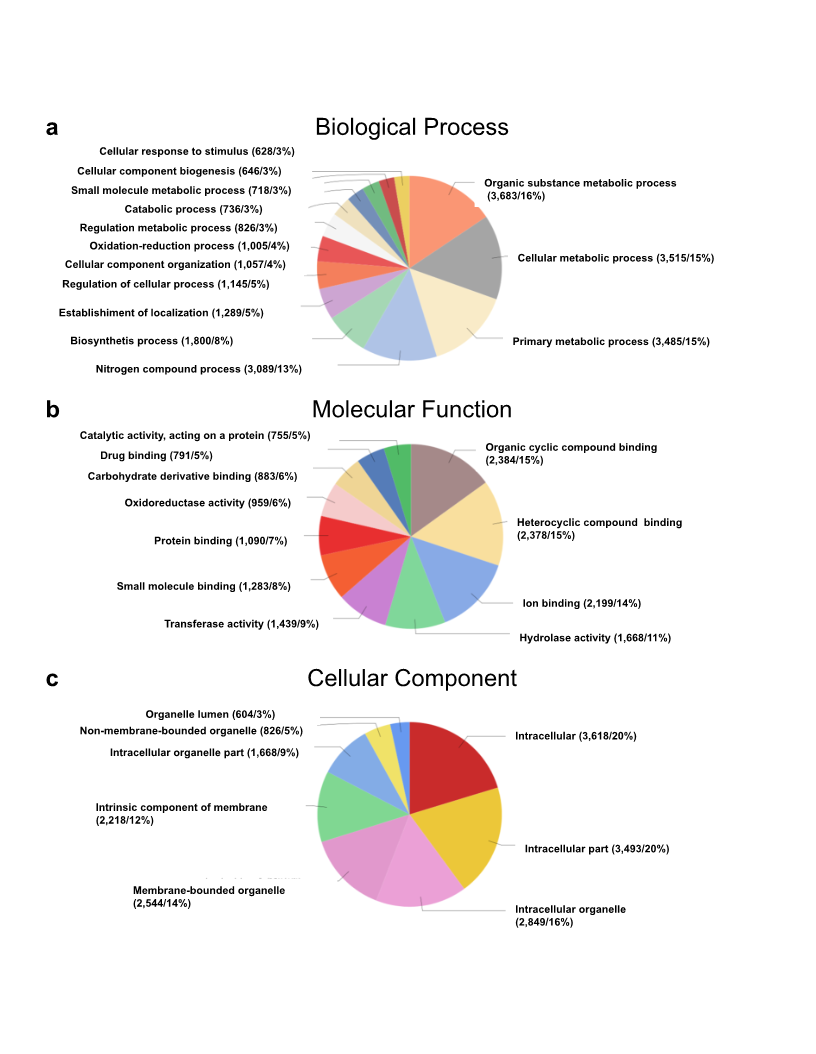

Supplement: evz207_Supplementary_Data [file evz207_supplementary_data.zip › De Miccolis Angelini et al. 2019 supplementary fig. S2.tiff]

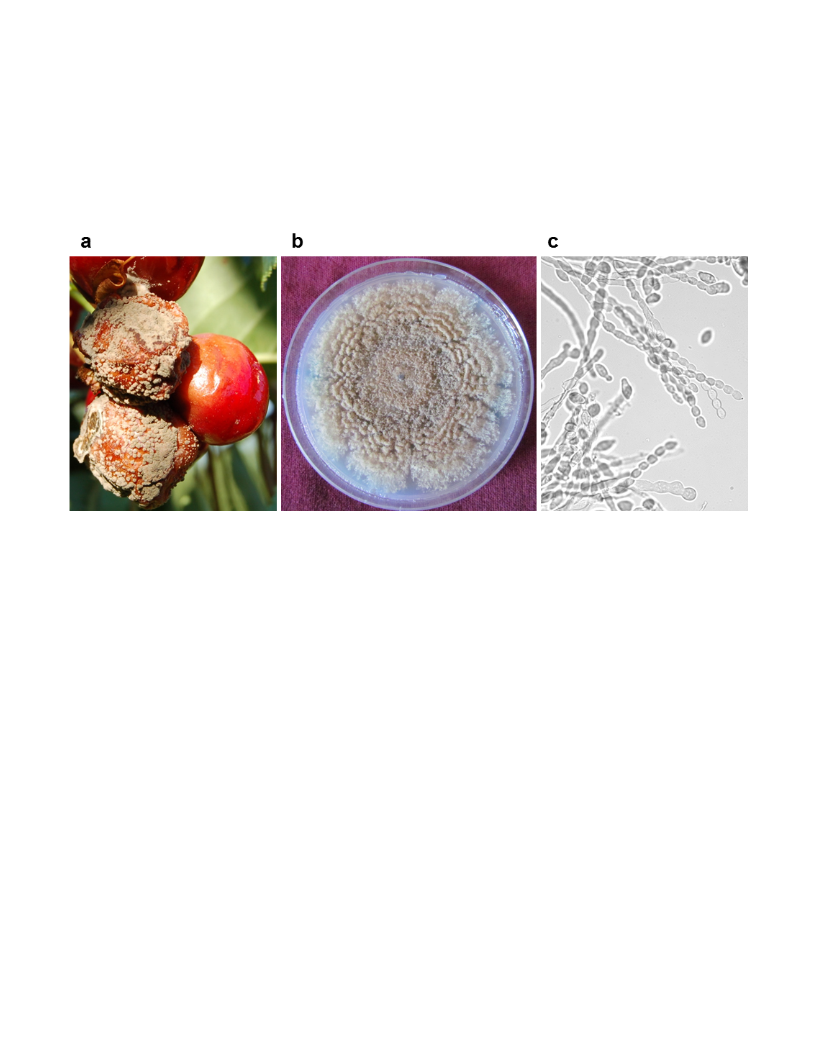

Supplement: evz207_Supplementary_Data [file evz207_supplementary_data.zip › De Miccolis Angelini et al., 2019. supplementary fig. S1.tiff]
